# Medical image encryption algorithm based on a new five-dimensional multi-band multi-wing chaotic system and QR decomposition

**DOI:** 10.1038/s41598-023-50661-9

**Published:** 2024-01-03

**Authors:** Zeben Zhuang, Zhiben Zhuang, Tao Wang

**Affiliations:** 1 Department of Critical Care Medicine, People’s Hospital of Fengjie, Fengjie, 404600 Chongqing China; 2 School of Mathematics and Computational Science and Key Laboratory of Intelligent Control Technology for Wuling-Mountain Ecological Agriculture in Hunan Province, Huaihua University, Huaihua, 418000 Hunan China; 3 College of Intelligent Systems Science and Engineering, Hubei Minzu University, Enshi, 445000 Hubei China

**Keywords:** Medical research, Mathematics and computing, Physics

## Abstract

In this study, we propose a medical image encryption algorithm based on a new five-dimensional (5D) multi-band multi-wing chaotic system and QR decomposition. First, we construct a new 5D multi-band multi-wing chaotic system through feedback control, which has a relatively large Lyapunov exponent. Second, we decompose the plaintext image matrix and chaotic sequence into an orthogonal matrix and upper triangular matrix using QR decomposition. We multiply the orthogonal matrix decomposed from the original image by the orthogonal matrix decomposed from the chaotic sequence. In this process, we use the chaotic sequence to control left and right multiplication. Simultaneously, we chaotically rearrange the elements in the upper triangular matrix using the improved Joseph loop and then multiply the two resulting matrices. Finally, we subject the product matrix to bit-level scrambling. From the theoretical analysis and simulation results, we observed that the key space of this method was relatively large, the key sensitivity was relatively strong, it resisted attacks of statistical analysis and gray value analysis well, and it had a good encryption effect for medical images.

## Introduction

With the rapid development of technology and medical conditions, medical images are spreading rapidly on networks, which is resulting in great hidden dangers to the privacy of patients. Therefore, it is very important to construct better medical image encryption algorithms.

At present, the construction of new chaotic systems is mainly achieved using feedback control^1–14^. In 2018, Liu et al. constructed a simple four-dimensional (4D) chaotic system by adding a feedback controller and applied it to medical image encryption^1^. In 2014 and 2015, Peng et al.^2^ and Liu^3^ constructed a new 4D hyperchaotic system by adding feedback controllers. The constructed chaotic system has a simple structure and complex dynamic characteristics. In 2020, Zhuang et al. constructed a new five-dimensional (5D) hyperchaotic system by adding a feedback controller. The system has a simple structure and can generate multiple rings and multiple wings in multiple directions^4^. In 2022, Lai et al. proposed the construction of a new hyperchaotic system by introducing the nonlinear function sin(*x*)^6^. In 2018, Liu et al. proposed the construction of a new chaotic system by introducing the nonlinear function sinh(*x*)^9^. In 2022, Liu et al. proposed the construction of a new *n*-dimensional conservative chaotic system by adding a feedback controller^10^. In 2022, Zheng et al. proposed the construction of a new chaotic system by introducing the nonlinear function cos(*x*)^11^. In 2021, Liang et al. proposed the construction of a new 5D chaotic system by adding a feedback controller^14^. Although the chaotic systems proposed in Refs.^5^ and^14^ have a simple structure and complex dynamic characteristics, they do not have multi band characteristics. Moreover, compared with this paper, the maximum Lyapunov exponent of the system equations is not large enough.

With the development of chaos theory and its application, experts and scholars have proposed many medical image encryption algorithms based on chaos. Compared with the traditional medical image encryption algorithm, the medical image encryption algorithm based on chaos has higher security, stronger anti-attack ability, and good characteristics in terms of complexity^15^. Therefore, experts and scholars have proposed many medical image encryption algorithms based on chaos^1,7–9,13,14,16–23^. In 2019, Belazi et al.^8^ proposed the use of the SHA-256 hash function and DNA coding for medical image encryption. In 2017, Cao et al.^16^ proposed the generation of various edge images through various thresholds and edge detectors, and then encrypted each edge image. In 2021, Kamal et al.^18^ introduced the use of jagged patterns, rotation, and random arrangement to scramble image blocks. In 2016, Dai et al.^19^ proposed encryption in the upper four bits and kept the lower four bits unchanged to improve the efficiency of medical image encryption. In 2020, Bi et al.^20^ applied an adaptive function to medical image encryption, which improved the complexity of encryption technology. In 2020, Zhuang et al. proposed the QR decomposition method to decompose the plaintext image matrix and five chaotic sequences into an orthogonal matrix and upper triangular matrix, respectively. Then they multiplied the orthogonal matrix decomposed from the plaintext image matrix by the five orthogonal matrices decomposed from the five chaotic sequences^4^. In the process of multiplication, only the orthogonal matrix decomposed from the plaintext image was left multiplied, and compared with using chaotic sequences to control left and right multiplication, randomness was poor.

Based on appeal analysis, we construct a new 5D chaotic system by adding two feedback controllers *w* and *v*, and introduce the nonlinear function sin(*x*) into the system. Theoretical analysis and simulation experiments demonstrated that the system had a relatively large Lyapunov exponent, and generated multiple bands and multiple wings in multiple directions. Therefore, compared with the traditional chaotic system, the proposed system achieved better randomness and complexity. In terms of encryption technology, we use QR decomposition to decompose the plaintext image matrix and chaotic sequence into orthogonal matrices and upper triangular matrices. We multiply the orthogonal matrix decomposed from the original image by the orthogonal matrix decomposed from the chaotic sequence. In this process, we use the chaotic sequence to control left and right multiplication. Compared with Ref.^4^, this algorithm achieved better randomness. Simultaneously, we chaotically rearrange the elements in the upper triangular matrix using the improved Joseph loop and then multiply the two resulting matrices. Through these treatments, the difficulty of exhaustive attacks increases. All attack methods based on explicit plaintext ciphertext mapping, except exhaustive attacks, are ineffective for this method and have high security.

## Improved Joseph traversal mapping

Joseph's problem describes $n$ individuals in a circular order. Counting from the first person, the *m*th person is eliminated repeatedly until only one person remains^24^.

According to the Joseph problem, we can uniquely determine an arrangement consisting of $n$ elements in the order of elimination. Given $n$ and $m$, we can obtain a unique permutation of (1,2,…, $n$) denoted by $f_{ysf} (n,m)$. For example, $f_{ysf} (7,3)$ corresponds to the sequence 3, 6, 2, 7, 5, 1, 4; that is, the mapping $f_{ysf} (7,3)$ yields the permutation 3, 6, 2, 7, 5, 4, 1.

To increase the permutation variables generated by the Joseph traversal map, the Joseph loop is applied with an interval constraint, which makes the generated permutation irregular. The corresponding Joseph traversal map is denoted by $f_{ysf}^{1} (n,q,m)$.

## New five-dimensional multi-band multi-wing chaotic system

The famous Lorenz system is as follows:

1 $$\left\{ \begin{gathered} \mathop x\limits^{.} = - 10(x - y), \hfill \\ \mathop y\limits^{.} = - xz + 28x - y, \hfill \\ \mathop z\limits^{.} = xy - \frac{8}{3}z. \hfill \\ \end{gathered} \right.$$

Based on system (1), we introduce two controllers $w$ and $v$, and make $w$ feedback to the original controller $x$, $v$ feedback to the original controller $y$, and the original controller $y$ and $z$ feedback to the new controller $w$. These three operations can make the five controllers of the system interact with each other, which increases the complexity of the relationship. The newly constructed 5D multi-band multi-wing chaotic system is as follows:

2 $$\left\{ \begin{gathered} \mathop x\limits^{.} = ay - bx - c\sin (w), \hfill \\ \mathop y\limits^{.} = dx - exv^{2} , \hfill \\ \mathop z\limits^{.} = fxy - gz, \hfill \\ \mathop w\limits^{.} = hyz - iw, \hfill \\ \mathop v\limits^{.} = jw - kv. \hfill \\ \end{gathered} \right.$$

where *a*, *b*, *c*, *d*, *e*, *f*, *g*, *h*, *i*, *j*, and *k* are the control parameters of the system.

### Lyapunov exponential spectrum analysis

**Figure 1:**
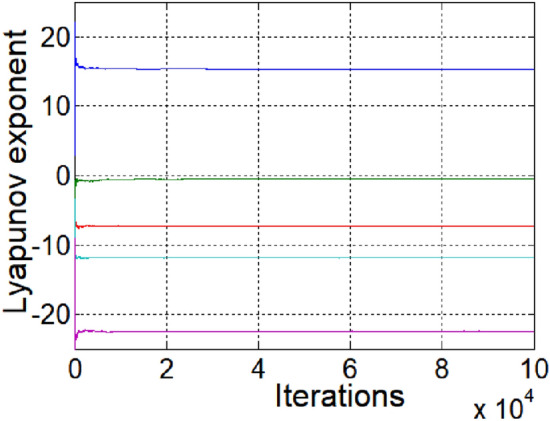
Lyapunov exponent diagram.

When the system parameters are $a = 10,b = 11,c = 1,d = 31,e = 1,f = 1,g = \frac{11}{3},h = 1,$ $i = 3,j = 1,$ and $k = 9$, the Lyapunov exponent spectrum of the system is as shown in Fig. 1. Figure 1 shows that the maximum Lyapunov exponent of the system is approximately 15, which indicates that the system has good randomness and complexity.

### Analysis of the five-dimensional multi-band multi-wing chaotic phase diagram

**Figure 2:**
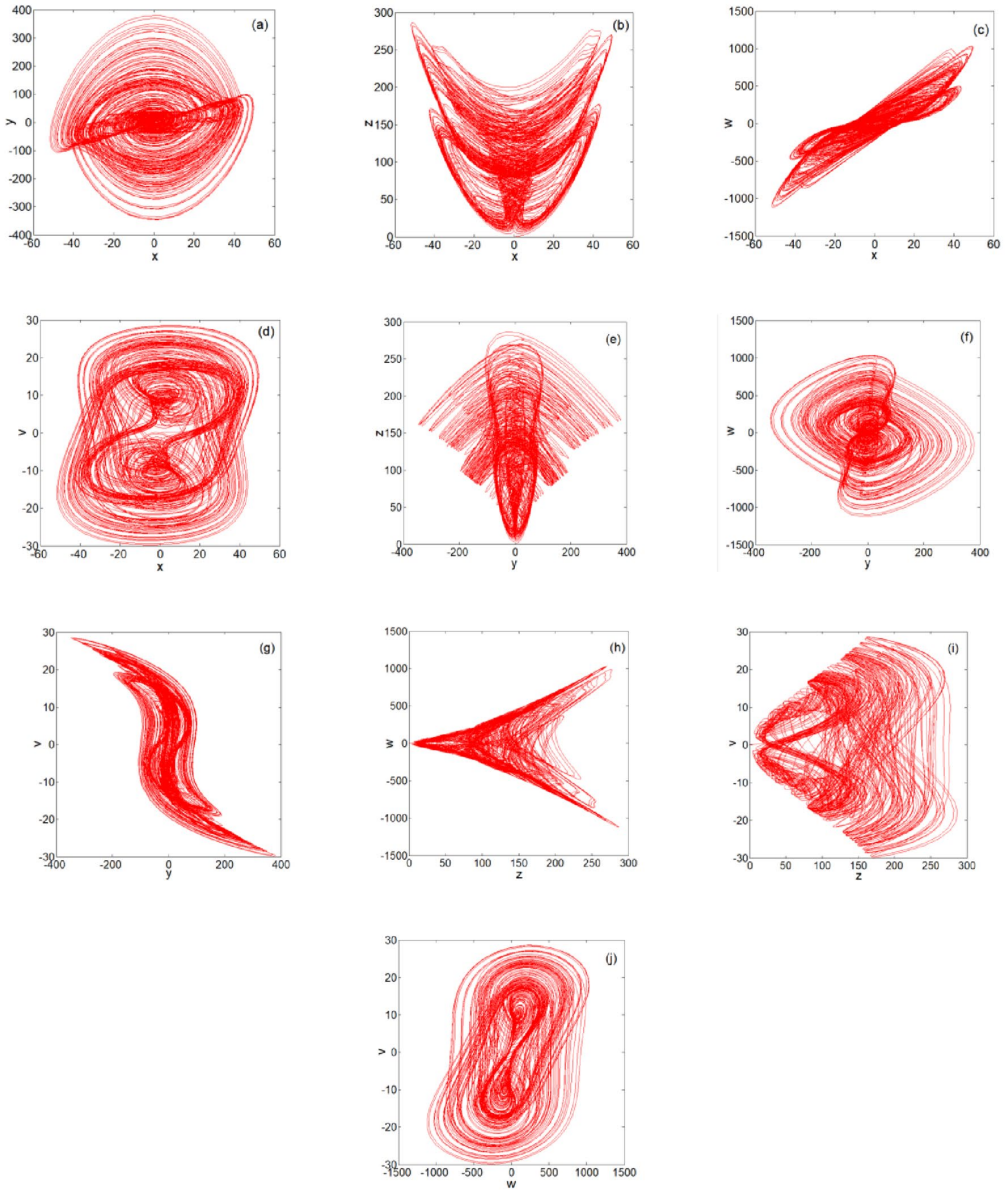
Plane phase diagrams: (**a**) *x*–*y* plane phase diagram; (**b**) *x*–*z* plane phase diagram; (**c**) *x*-*w* plane phase diagram; (**d**) *x*-*v* plane phase diagram; (**e**) *y*–*z* plane phase diagram; (**f**) *y*-*w* plane phase diagram; (**g**) *y*-*v* plane phase diagram; (**h**) *z*-*w* plane phase diagram; (**i**) *z*-*v* plane phase diagram; and (**j**) *w*-*v* plane phase diagram.

**Figure 3:**
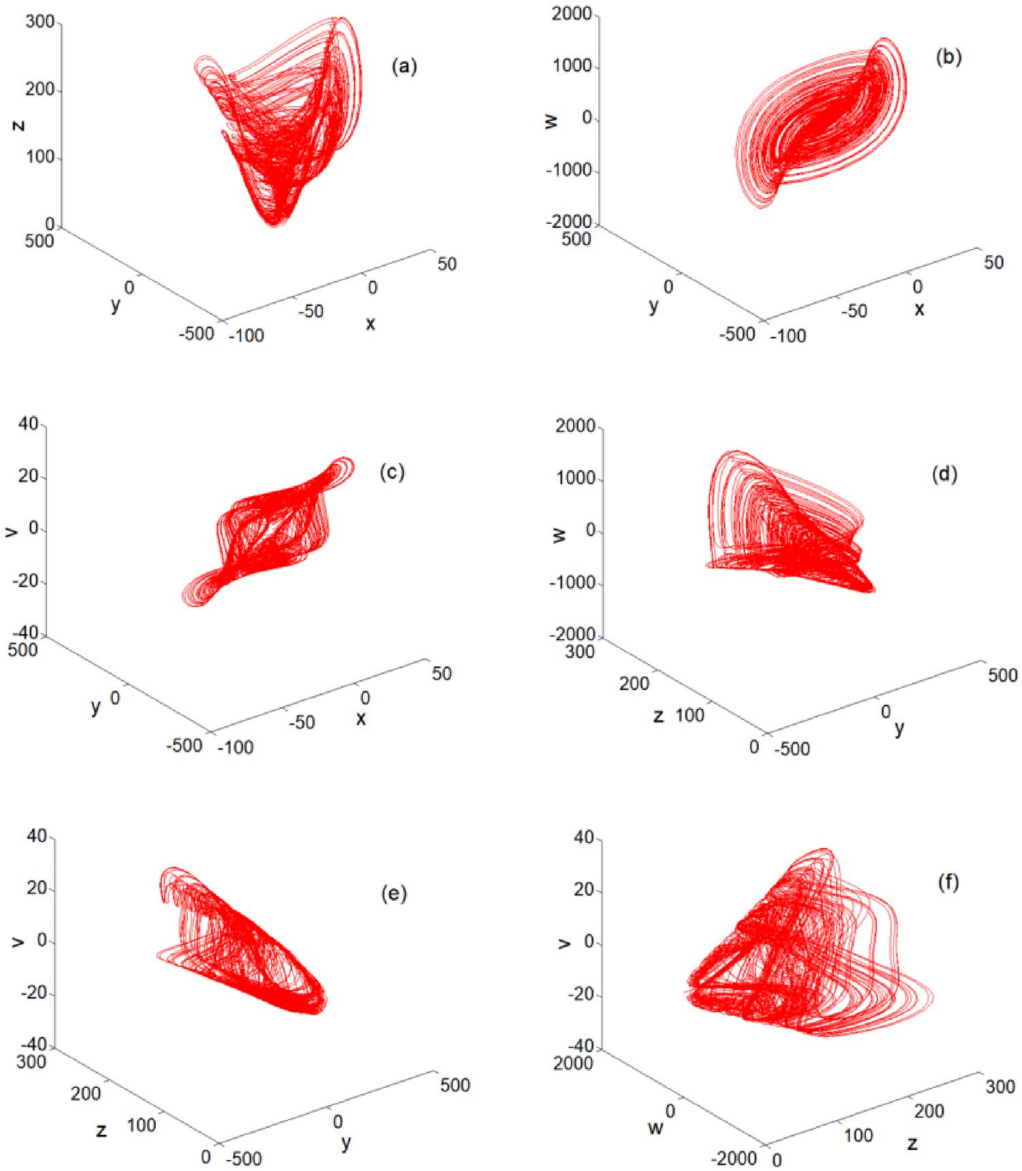
3D space phase diagrams: (**a**) *x*–*y*-*z* space phase diagram; (**b**)* x*–*y*-*w* space phase diagram; (**c**)* x*–*y*-*v* space phase diagram; (**d**)* y*–*z*-*w* space phase diagram; (**e**)* y*–*z*-*v* space phase diagram; and (**f**) *z*-*w*-*v* space phase diagram.

When the system parameters are $a = 10,b = 11,c = 1,d = 31,e = 1,f = 1,g = \frac{11}{3},h = 1,$ $i = 3,j = 1$, and $k = 9$, the plane phase diagram of the multiple bands and multiple wings generated by Eq. (2) is as shown in Fig. 2. The three-dimensional (3D) phase diagram of the multiple bands and multiple wings generated by system (2) is shown in Fig. 3. Figures 2 and 3 clearly show that the chaotic system can generate multiple bands and multiple wings in multiple directions.

### Two-dimensional time series diagram

**Figure 4:**
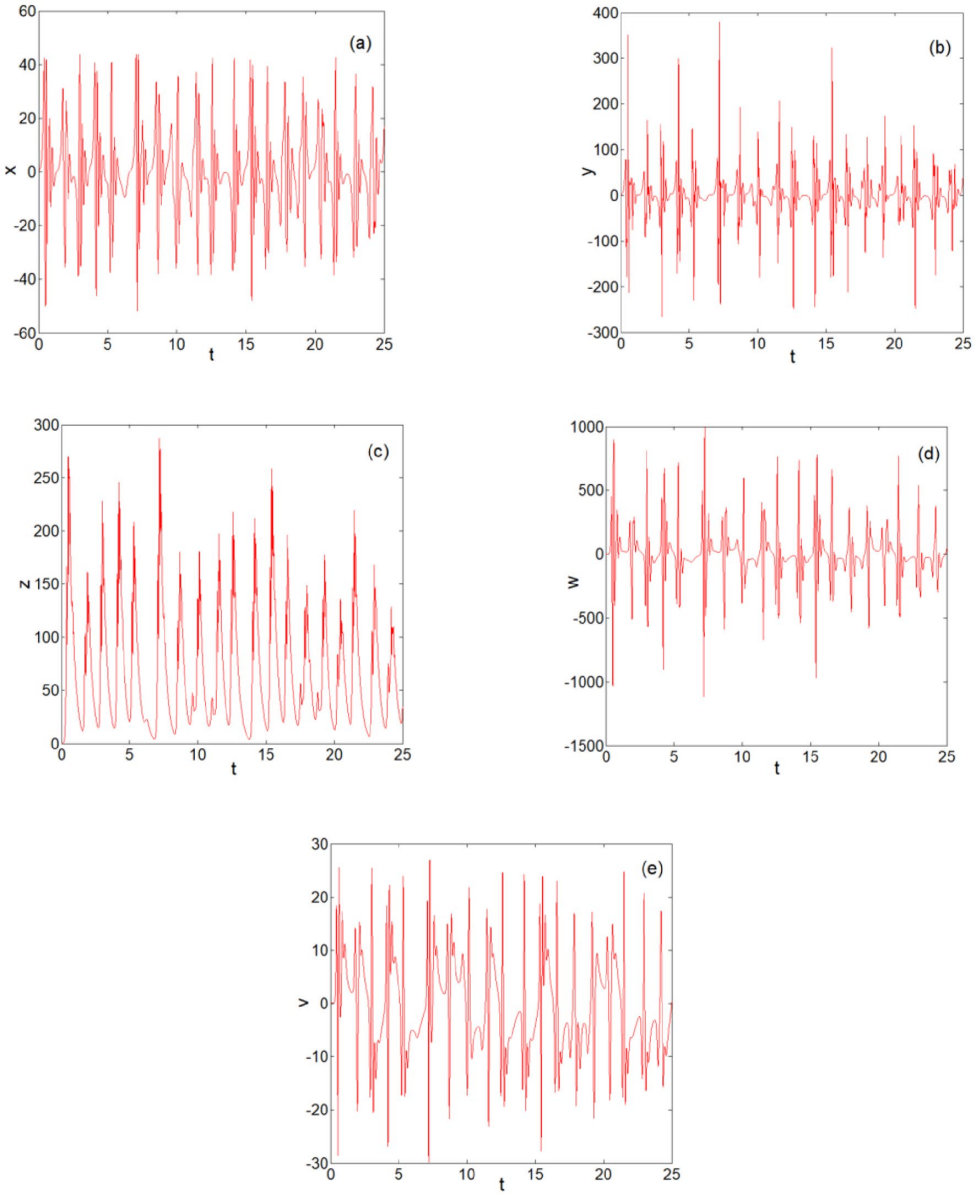
Two-dimensional time series diagrams: (**a**) *t*-*x* two-dimensional time series diagram; (**b**) *t*-*y* two-dimensional time series diagram; (**c**) *t*-*z* two-dimensional time series diagram; (**d**) *t*-*w* two-dimensional time series diagram; and (**e**) *t*-*v* two-dimensional time series diagram.

For the system parameters $a = 10,b = 11,c = 1,d = 31,e = 1,f = 1,g = \frac{11}{3},h = 1,i = 3,$ $j = 1$, and $k = 9$, Fig. 4 shows the sequence diagram of *x*, *y*, *z*, *w*, and *v* changing with time* t*. We can clearly observe from Fig. 4 that system (2) is in a chaotic state.

### NIST SP800-22 test

We used the National Institute of Standards and Technology (NIST) SP800-22 test to verify that the output sequence of a chaotic system is better than normal pseudo-randomness. NIST SP800-22 has 15 subtests, each of which yields a corresponding *P*-value, and proves that the chaotic system passes the NIST test when all *p*-values are greater than 0.01. The results of the tests are listed in Table 1. Clearly, all the *P*-values in the table are within the desired range, which indicates that the proposed chaotic system has high chaotic performance.

**Table 1:** Results of the SP800-22 tests.

Test name	P-value	Result
NIST test results		
Approximate entropy	0.3549	Pass
Block frequency	0.4303	Pass
Cumulative sums (forward)	0.6848	Pass
Cumulative sums (reverse)	0.9581	Pass
FFT	0.4506	Pass
Frequency	0.9965	Pass
Linear complexity	0.0650	Pass
Longest run of ones	0.1067	Pass
Non-overlapping template	0.6423	Pass
Overlapping template	0.7682	Pass
Random excursions	0.7999	Pass
Random excursions variant	0.1199	Pass
Rank	0.012	Pass
Runs	0.5606	Pass
Serial test	0.1406	Pass
Universal	0.1998	Pass

### Chaotic decision tree algorithm

Compared with many classic algorithms for detecting chaotic behavior, the chaotic decision tree algorithm is a more convenient and faster algorithm^25,26^. We tested the proposed system on the chaos decision tree algorithm. The output results are presented in Table 2, which indicates that chaotic behavior is inherent in system (2).

**Table 2:** Test results and comparative analysis.

Map		Permutation entropy	Chaos/stochastic/periodic	*K*
Proposed scheme	*x*	5.4752	Chaotic	0.9911
	*y*	5.0583	Chaotic	0.9823
	*z*	5.0751	Chaotic	0.9917
	*w*	6.0012	Chaotic	0.9965
	*v*	5.0117	Chaotic	0.9833
Umbrella map	*x*	5.3492	Chaotic	0.9825
	*y*	6.0661	Chaotic	0.9956
Henon map	*x*	3.0864	Periodic	0.7524
	*y*	3.0573	Periodic	0.7484
Logistic map		5.0497	Chaotic	0.9974

## Algorithm introduction

### Encryption method

**Figure 5:**
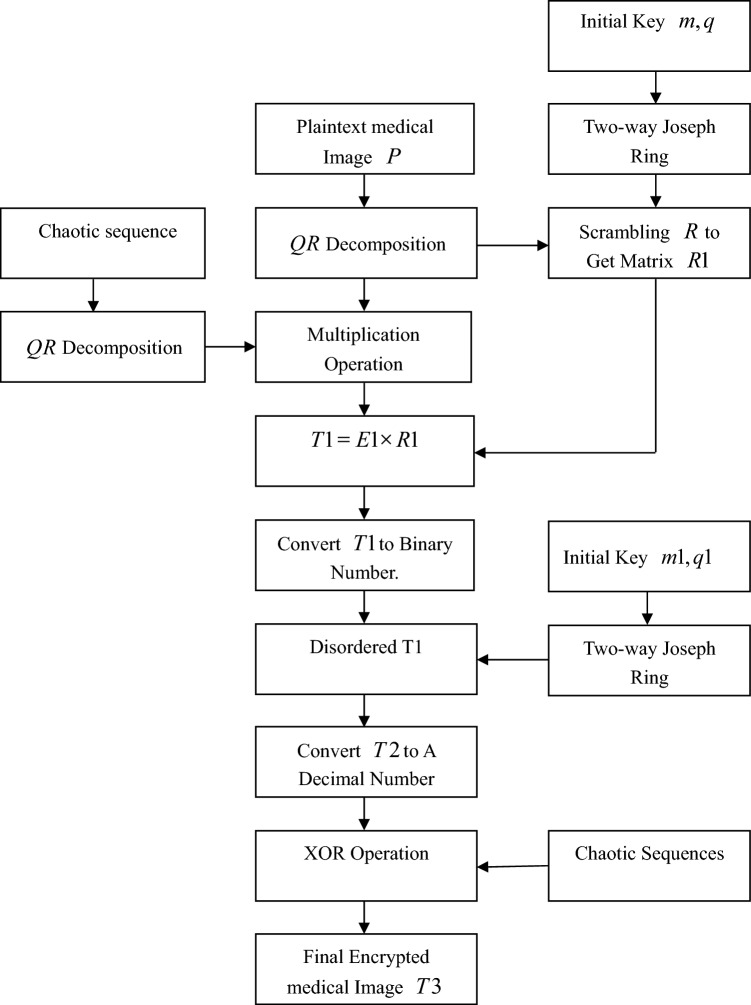
Encryption flow chart.

Given an $M \times N$ grayscale medical image $P$, the encryption scheme is as follows, which is shown in Fig. 5:

**Step 1:** Input the initial value of Eq. (2)$y_{1} { = [}x_{0} {,}y_{0} {,}z_{0} {,}w_{0} {,}v_{0} {]}$ and $x_{1}^{0} ,x_{2}^{0} ,x_{3}^{0} ,x_{4}^{0} ,x_{5}^{0}$. Call the ode45 function.

**Step 2:** Iterate system (2) $N1$ times to avoid the transient influence of the initial value on the system. Continue iterating $M \times N$ times to obtain five sequences $b1,b2,b3,b4,b5$. The formula for calculating $N1$ is as follows:

3 $$N1 = 200 + \bmod \left( {\left( {\left( {\sum\limits_{j = 1}^{5} {x_{j}^{0} } } \right) - floor\left( {\sum\limits_{j = 1}^{5} {x_{j}^{0} } } \right)} \right) \times 10^{7} ,200} \right),$$

where $floor(e)$ is on the largest integer less than $e$. Because of the poor randomness of the starting value of the chaotic sequence, the value is taken after 200 values of the chaotic sequence.

**Step 3:** Treat chaotic sequence $bj$ as follows:

4 $$bi = round(\bmod (bj((N1 + 1):M \times N) \times 10^{4} ,256)),(i,j = 1,...,5),$$

where $bj(g:h)$ is the sequence of the $gth$ value to the $hth$ value in $bj$.

**Step 4:** Input a gray medical image $P$. Then decompose $P$ into an orthogonal matrix $E$ and an upper triangular matrix $R$ of size $M \times N$ using $QR$ decomposition.

**Step 5:** Decompose chaotic sequences into *n* orthogonal matrices and *n* upper triangular matrices through QR decomposition, denoted by $$E_{1} ,E_{2} ,$$$$...,E_{n}$$. Then, use the chaotic sequence to control whether *E*_*i*_ (*i* = 1,…,*n*) and *E* are left multiplied or right multiplied. Denote the resulting matrix by $$E1$$, that is, $$E1 = E_{il} ...E_{i1} EE_{j1} ...E_{jk} ,(k + l = n)$$.

**Step 6:** Convert the upper triangular matrix $R$ into a matrix with one row and $M \times N$ columns. Input the initial key $m,q$ for the bidirectional Joseph ring. Disturb the elements of $R$ using the bidirectional Joseph ring. Then convert $R$ into an $M \times N$ matrix, which is the disturbed matrix denoted by $R1$.

**Step 7:** Multiply $E1$ and $R1$ to obtain the matrix $P1 = E1 \times R1$.

**Step 8:** Convert $P1$ into matrix $P2$ with one row and $M \times N$ columns. Then map each element in $P2$ to a value between 0 and 255. The mapping formula is as follows:

5 $$round\left( {\frac{P1(i) - (\min (P1) - 1)}{{\max (P1) - (\min (P1) - 1)}} \times 255} \right),$$

where $round(f)$ denotes the rounding of $f$.

**Step 9:** Convert $P2$ into a binary number to obtain matrix $P2{\prime}$. Then convert $P2{\prime}$ into matrix $P3$ with one row and $M \times N \times 8$ columns.

**Step 10:** Input the initial key $m1,q1$ for the bidirectional Joseph ring. Disturb all the elements in $P3$ using the bidirectional Joseph ring. Convert the disturbed matrix $P3$ into matrix $P4$ of size $(M \times N) \times 8$.

**Step 11:** Convert the elements of $P4$ into decimal numbers and convert the resulting matrix into matrix $P5$ with one row and $M \times N$ columns.

**Step 12:** Perform a bitwise XOR operation on $P5$ and $b4$. A new sequence $P6$ is obtained, which converts $P6$ into matrix $P7$ of size $M \times N$. $P7$ is the final encrypted image.

### Decryption algorithm

**Step 1:** Input $P7$ and the key $y_{1} { = [}x_{0} {,}y_{0} {,}z_{0} {,}w_{0} {,}v_{0} {]},x_{1}^{0} ,x_{2}^{0} ,x_{3}^{0} ,x_{4}^{0} ,x_{5}^{0}$. Perform a bitwise XOR operation on $P7$ and the chaotic sequence generated by system (2) to obtain matrix $P8$.

**Step 2:** Convert $P8$ into a binary number and enter the key $m1,q1$. The binary number is restored using a bidirectional Joseph ring to obtain matrix $P9$.

**Step 3:** Convert $P9$ into a decimal number to obtain matrix $P10$. Then reduce $P10$ to $P1$ using the following formula:

6 $$(P10(i) \div 255) \times (\max (P1) - (\min (P1) - 1)) + (\min (P1) - 1).$$

**Step 4:** Multiply by the inverses of $E_{il} ,...,E_{i1} ,E,E_{j1} ,...,E_{jk}$ on the left side of $P1$ to obtain matrix $R1$.

**Step 5:** Convert $R1$ into $M \times N$ columns and enter the key $m,q$. Reduce the $M \times N$ columns using a bidirectional Joseph ring to obtain matrix $P11$.

**Step 6:** Convert $P11$ into a matrix of size $M \times N$, that is, matrix $R$, and multiply by $E$ and $R$ to obtain the decrypted image $P$.

## Results and safety analysis

### Experimental platform

Experiments were conducted on a PC configured with an Intel (R) Core (TM) i5-9400F CPU running at 2.90 GHz with 16 GB memory and a Windows 10 64-bit operating system. The above encryption algorithm was implemented using MATLAB R2014a.

### Experimental results

**Figure 6:**
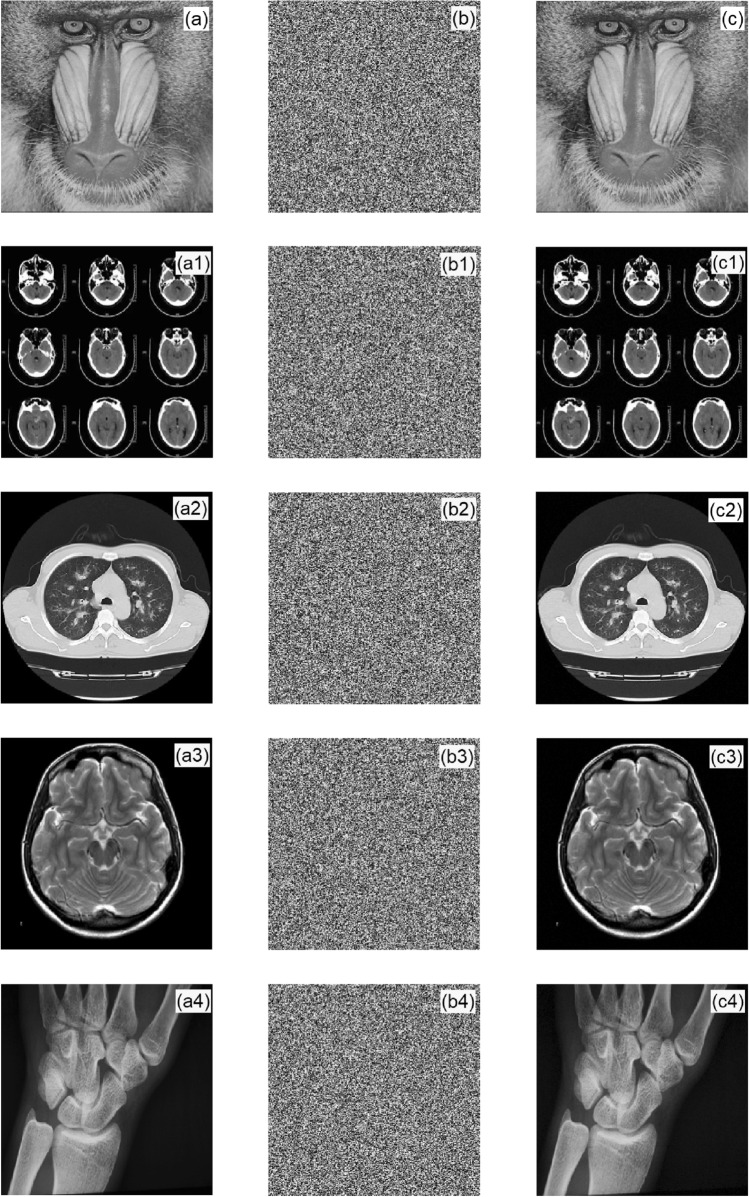
Simulation result diagrams: (**a**,**a1**,**a2**,**a3**,**a4**) are the original images; (**b**,**b1**,**b2**,**b3**,**b4**) are the encrypted images; and (**c**,**c1**,**c2**,**c3**,**c4**) are the decrypted images.

In the simulation experiment, Baboon, brain CT, chest CT, DR film, and MRI gray images were selected, and their pixels were 256 × 256. Figure 6 shows the original image, ciphertext image, and decrypted image.

### Information entropy

Information entropy is an important factor used to evaluate the randomness of encrypted images. The formula for solving information entropy is as follows:

7 $$L(n) = - \sum\limits_{j = 1}^{K} {p(n_{j} )} \log_{2} p(n_{j} ),$$

where $p(n_{j} )$ is the probability of $n_{j}$ and $K$ is the total quantity. For grayscale encrypted images, 8 is the maximum theoretical value of information entropy. The information entropy of Baboon, brain CT, chest CT, DR film, and MRI after encryption and the test values from the literature^11,29–34^ are shown in Table 3.

**Table 3:** Information entropy test values and comparative analysis.

Image	Baboon	Brain CT	Chest CT	DR film	MRI
Ciphertext	7.9976	7.9975	7.9973	7.9969	7.9972
Literature^11^	7.9974	–	–	–	–
Literature^29^	7.9974	–	–	–	–
Literature^30^	7.9865	–	–	–	–
Literature^31^	7.9609	–	–	–	–
Literature^32^	7.9672	–	–	–	–
Literature^33^	7.9938	–	–	–	–
Literature^34^	7.9975	–	–	–	–

The test values for information entropy demonstrated that this algorithm had a good encryption effect.

### Key space analysis

The size of the key space is an important factor for the strength of the image encryption algorithm. The initial key of the proposed method is composed of the initial values $y_{1} { = [}x_{0} {,}y_{0} {,}z_{0} {,}w_{0} {,}v_{0} {]}$ and system parameters *a*, *b*, *c*, *d*, *e*, *f*, *g*, *h*, *i*, *j*, and *k*. The key space of the algorithm is 10^240^. If the key space of an image encryption algorithm is greater than 2^100^, it is safe^27,28^. Because 10^240^ is far greater than 2^100^, the proposed method is sufficiently safe.

### Histogram analysis

**Figure 7:**
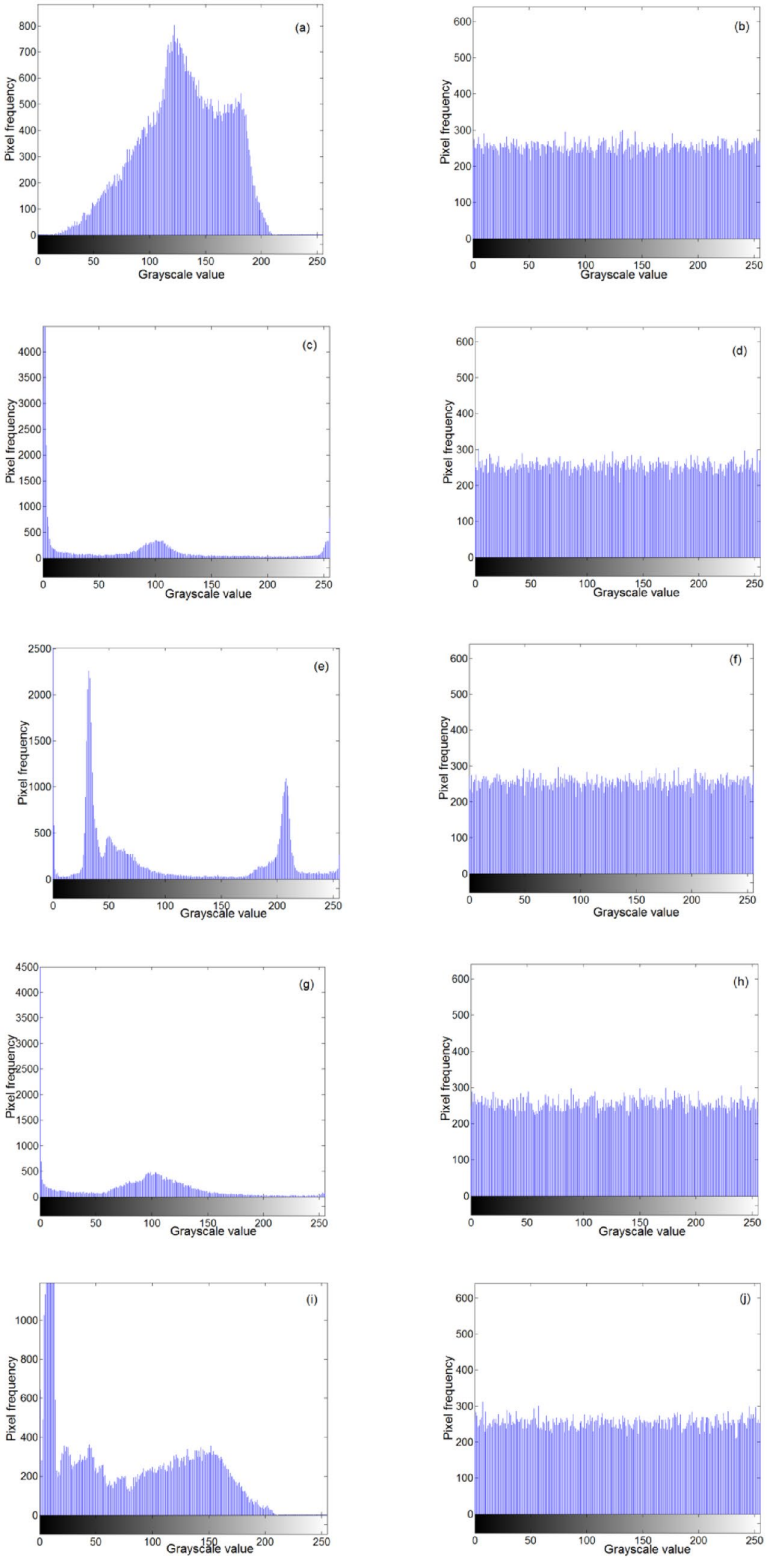
Histograms: (**a**,**c**,**e**,**g**,**i**) are the histograms of the plaintext images; and (**b**,**d**,**h**,**f**,**j**) are the histograms of the encrypted images.

A histogram reflects the distribution of image pixel values. The flatter the histogram, the more uniform the distribution of pixel values. The histograms of the Baboon, brain CT, chest CT, DR film, and MRI grayscale images are shown in Fig. 7. Figure 7 shows that the histogram distribution of the encrypted image was relatively uniform.

### Fixed-point ratio and average change value of the grayscale

The fixed-point ratio is the percentage of pixels whose grayscale value does not change after the image encryption of all pixels. It can be obtained using

8 $$F(I,J) = \frac{{\sum\nolimits_{k = 1}^{M} {\sum\nolimits_{l = 1}^{N} {g(k,l)} } }}{MN} \times 100\% ;$$

where $$g(k,l) = \left\{ \begin{gathered} 1,f_{kl} = c_{kl} \hfill \\ 0,f_{kl} \ne c_{kl} \hfill \\ \end{gathered} \right.$$. Table 4 shows the values calculated using formula (9).

**Table 4:** Fixed-point ratio test results.

Image	Total number of pixels	Fixed point number	Fixed point ratio (%)
Baboon	65,536	255	0.39
Brain CT	65,536	269	0.41
Chest CT	65,536	255	0.39
DR film	65,536	240	0.37
MRI	65,536	264	0.40

The average grayscale level change value can better evaluate the degree of gray level change of the encrypted image and can be obtained using

9 $$E(J,I) = \frac{{\sum\limits_{k = 1}^{M} {\sum\limits_{l = 1}^{N} {\left| {c_{kl} - } \right.\left. {f_{kl} } \right|} } }}{MN}.$$

Table 5 shows the average change values of the gray level calculated using formula (10).

**Table 5:** Average gray level change.

Image	Baboon	Brain CT	Chest CT	DR film	MRI
Average change value of gray scale	69.8816	110.0441	99.1073	99.8431	91.3332

### Sensitivity analysis

**Figure 8:**
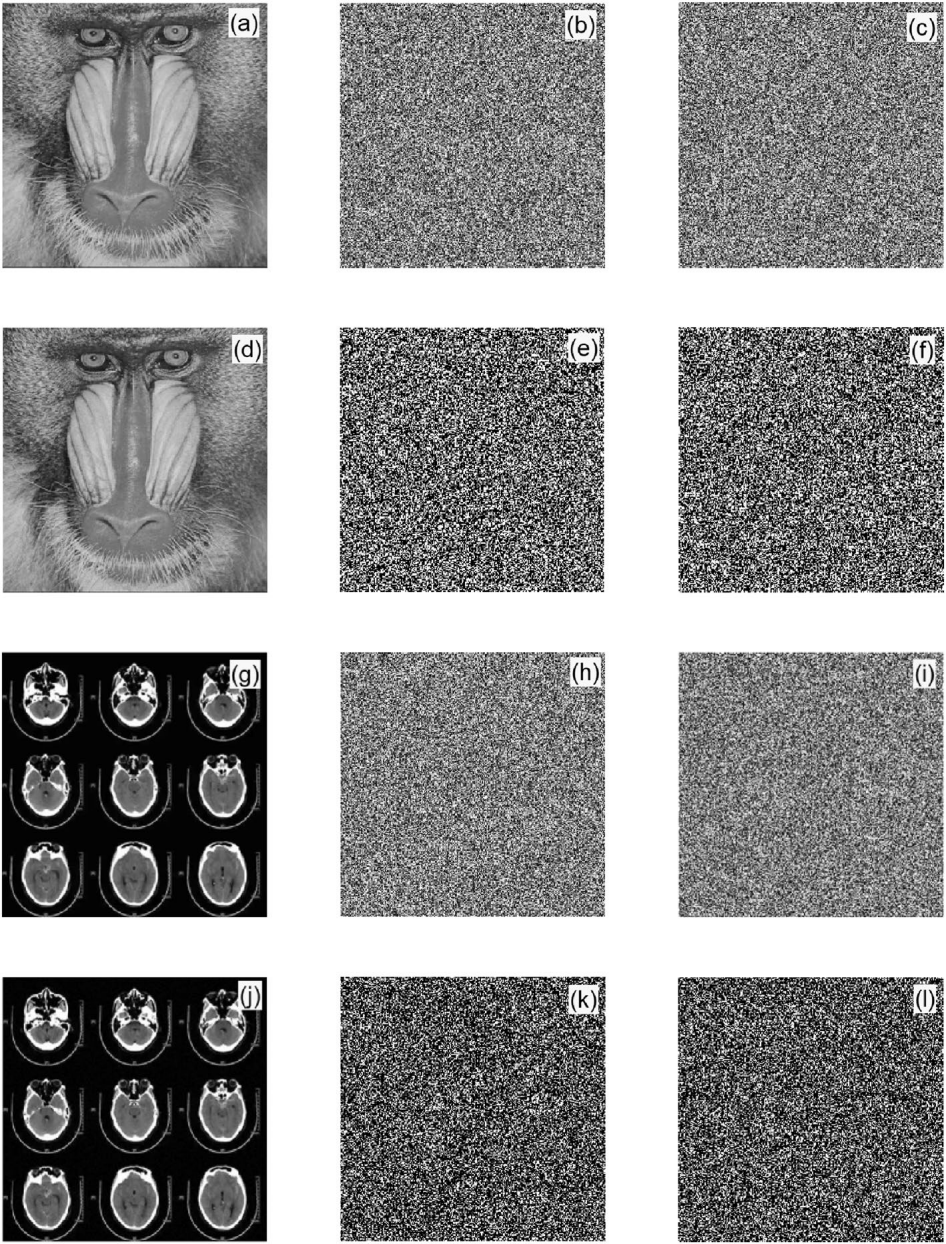
Key sensitivity analysis diagrams: (**a**) and (**g**) are the original images; (**b**,**c**,**h**,**i**) are the ciphertext images; and (**e**,**f**,**k**,**l**) are the misinterpreted ciphertext images.

Key sensitivity means that a small change in the initial key causes a great change in the ciphertext. In the experiment, classic Baboon and brain CT images were used as examples, as shown in Fig. 8. Figure 8a,g are the original images of Baboon and brain CT, respectively. The ciphertext images encrypted with the initial key $y_{1} = [0.5,0.2,0.7,0.5,0.1]$ are shown in Fig. 8b,h, respectively. The ciphertext images encrypted with the initial key $y_{2} = [0.5,0.2,0.7,0.5000000000000001,0.1]$ are shown in Fig. 8c,i, respectively. Figure 8e,k, respectively, show the misinterpretation diagrams decrypted with *y*_2_. Figure 8f,l, respectively, show the misinterpretation diagrams decrypted with *y*_1_.

Figure 8 show that the encryption method had good key sensitivity.

### Differential analysis

The more sensitive the ciphertext image to the plaintext image, the better the performance of the algorithm against differential attacks. To resist a differential attack, a good cryptosystem should ensure that any tiny modification in the plain-image should cause a significant difference in the cipher-image. The number of pixels change rate (*NPCR*) and unified average changing intensity (*UACI*) are typically used for differential attack analysis:

10 $${\text{NPCR}} = \frac{{\sum\limits_{i,j}^{M,N} {D\left( {i,j} \right)} }}{M \times N} \times 100\% ;$$

11 $${\text{UACI}} = \frac{{\sum\nolimits_{i,j}^{M,N} {\left| {C_{1} \left( {i,j} \right) - C_{2} \left( {i,j} \right)} \right|} }}{M \times N \times 255} \times 100\% .$$

where and are two cipher-images whose plaintext has only a *C*1 *C*2 different pixel, and *D*(*i*, *j*)is defined as

12 $$D(i,j) = \left\{ \begin{gathered} 1,C_{1} (i,j) \ne C_{2} (i,j) \hfill \\ 0,C_{1} (i,j) = C_{2} (i,j) \hfill \\ \end{gathered} \right.$$

The ideal values of *NPCR* and *UACI* are 99.61% and 33.46%, respectively. Table 6 shows a comparative analysis of the test values calculated by formulas (11) and (12), and the literature^5,29,30,33^.

**Table 6:** Key sensitivity comparative analysis.

Image	Baboon		Brain CT		Chest CT		DR film	
Index	NPCR	UACI	NPCR	UACI	NPCR	UACI	NPCR	UACI
Proposed	0.9961	0.3362	0.9960	0.3353	0.9957	0.3358	0.9966	0.3344
Literature^5^	0.9962	0.3344	–	–	–	–	–	–
Literature^29^	0.9966	0.3342	–	–	–	–	–	–
Literature^30^	0.9949	0.3216	–	–	–	–	–	–
Literature^33^	0.9911	0.3325	–	–	–	–	–	–

Table 6 shows that the encryption method had good key sensitivity.

### Correlation analysis

In this study, 3000 adjacent pixels were randomly selected from the plaintext and ciphertext images of Baboon, brain CT, chest CT, DR film, and MRI. The correlation coefficients of the original image and encrypted image in the horizontal direction, vertical direction, and diagonal direction can be calculated as follows:

12 $$E\left( n \right) = \frac{1}{M}\sum\limits_{j = 1}^{M} {n_{j} } ;$$

13 $$D\left( n \right) = \frac{1}{M}\sum\limits_{j = 1}^{M} {(n_{j} - E(n))^{2} } ;$$

14 $$Cov\left( {n,m} \right) = \frac{1}{M}\sum\limits_{j = 1}^{M} {(n_{j} - E(n))(m_{j} - E(m))} ;$$

15 $$r_{nm} = \frac{Cov(n,m)}{{\sqrt {D\left( n \right)} *\sqrt {D\left( m \right)} }},$$

where $m$ and $n$ are the grayscale values of two adjacent pixels in the image, and $M$ is the total number of pixels selected from the image.

**Figure 9:**
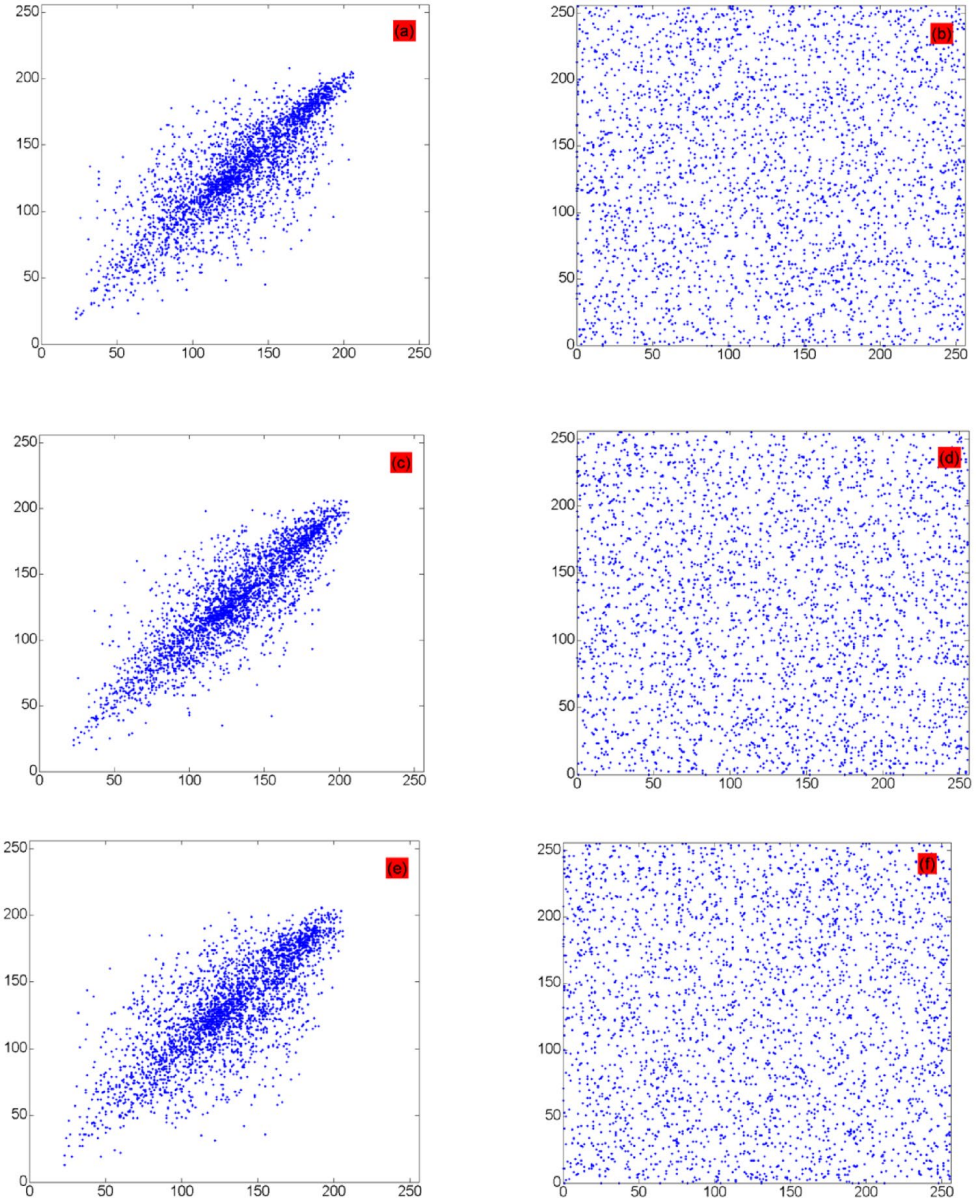
Correlation diagrams of Baboon: (**a**,**c**,**e**) are the pixel correlation diagrams of the original image in three directions; and (**b**,**d**,**f**) are the pixel correlation diagrams of the encrypted image in three directions.

**Figure 10:**
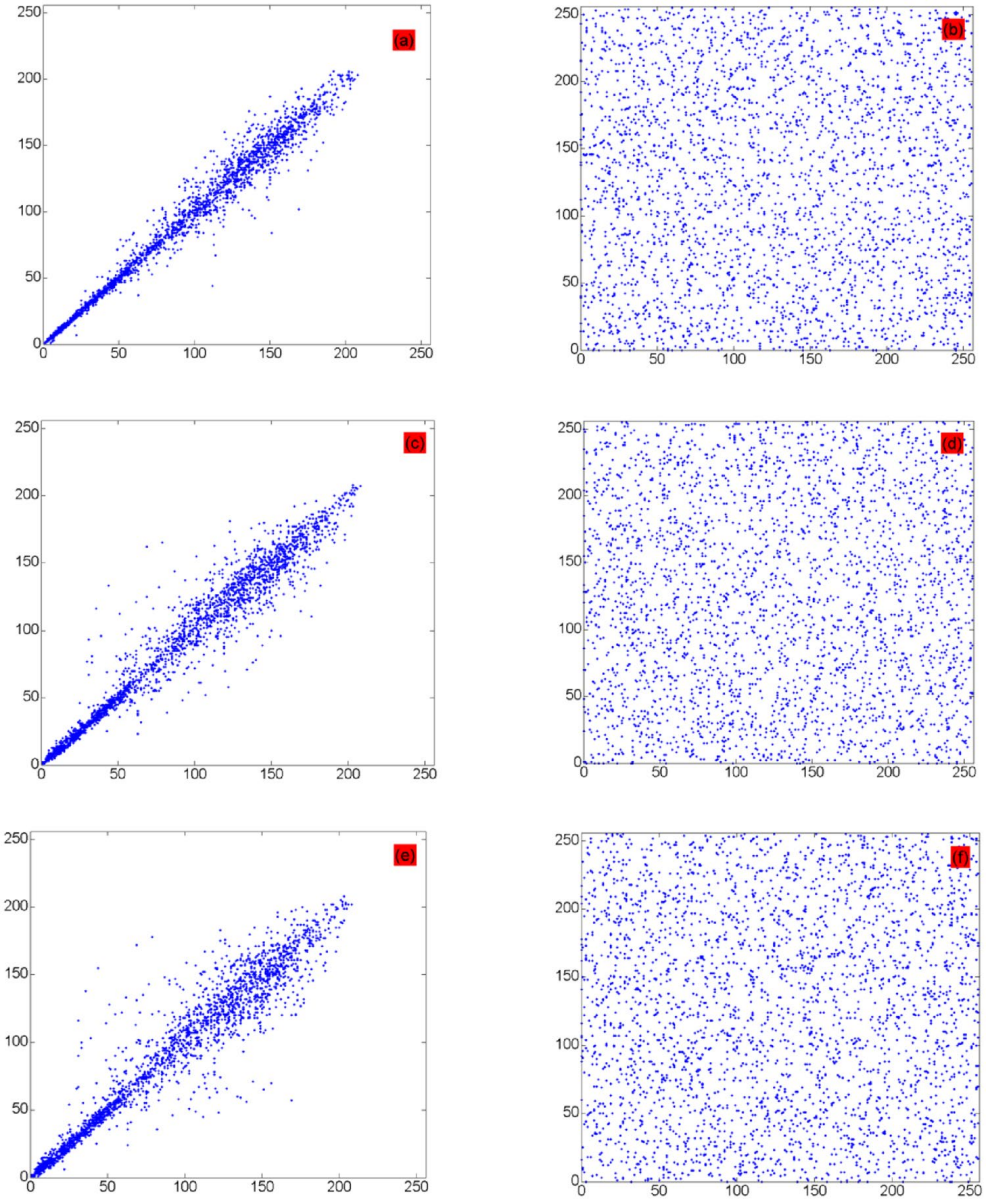
Correlation diagrams of DR film: (**a**,**c**,**e**) are the pixel correlation diagrams of the original image in three directions; and (**b**,**d**,**f**) are the pixel correlation diagrams of the encrypted image in three directions.

Table 7 shows the simulation results. The pixel correlation diagrams of the original and encrypted images of Baboon and DR film in the horizontal, vertical, and diagonal directions are shown in Figs. 9 and 10, respectively, which show that the correlation coefficients of the original image are close to 1, whereas those of the encrypted image are approximately 0 along all three directions. Table 7 shows that the adjacent pixels of the encrypted image have very low correlation. Thus, the proposed algorithm can effectively resist a statistical attack. The test results in Table 7, and Figs. 9 and 10 show that the proposed method broke the correlation between adjacent pixels well.

**Table 7:** Pixel correlation test results.

Image	Horizontal direction		Vertical direction		Diagonal direction	
	Plaintext	Ciphertext	Plaintext	Ciphertext	Plaintext	Ciphertext
Baboon	0.8355	− 0.0344	0.8651	0.0298	0.7968	− 0.0599
Brain CT	0.8965	0.0261	0.8513	− 0.0333	0.7926	0.0714
Chest CT	0.9485	− 0.0208	0.9794	− 0.0386	0.9454	− 0.0235
DR film	0.9611	0.0901	0.9454	0.0308	0.9206	− 0.0072
MRI	0.9917	− 0.0129	0.9853	0.0157	0.9790	− 0.0056

### Analysis of an anti-shear attack

**Figure 11:**
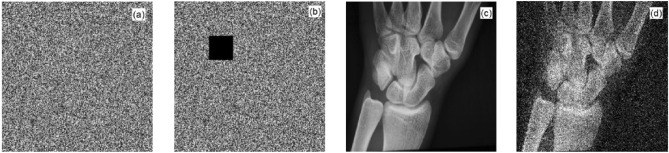
Anti-shear attack capability analysis chart: (**a**) ciphertext before cutting; (**b**) ciphertext after cutting; (**c**) decrypted image before cutting; and (**d**) decrypted image after cutting.

To test the anti-cropping ability of the algorithm, we cut out a 40 × 40 sized image from the middle of MRI's encrypted image, as shown in Fig. 11b, and then decrypted the cropped ciphertext image. The decrypted image is shown in Fig. 11d. Figure 11a shows the original encrypted image and Fig. 11c shows the decrypted image of the original encrypted image. Comparing Fig. 11c and d, we observed that the pixel values of some points in Fig. 11d had changed; however, the approximate information of the plaintext image was still displayed. Therefore, the encrypted images still had a certain decryption effect after being subjected to cropping attacks.

### Two-dimensional entropy analysis

Two-dimensional entropy reflects the spatial characteristics of the grayscale distribution of an image. Its calculation formula is as follows:

16 $$H(X) = - \sum\limits_{i} {\sum\limits_{j} {p(i,j)} } \log_{2} p(i,j).$$

The probability density function $p(i,j)$ is the grayscale co-occurrence matrix of adjacent pixel pairs, where ***i*** is the grayscale value of the pixel currently located at the center of a window and*** j*** is the grayscale value of the pixels adjacent to it in a specific order. The test results are shown in Table 8.

**Table 8:** Two-dimensional entropy test results.

Image	Baboon	Figure 5e	Figure 5h	Figure 5k	Figure 5n
Plaintext	7.3582	4.3347	6.3314	5.1188	7.2317
Ciphertext	7.9895	7.9897	7.9896	7.9888	7.9889

Table 8 shows that the encryption scheme had good encryption performance.

### Complexity comparison

The key space size of this algorithm is 10^240^, and the comparison results with Refs.^5,11,18,21,35,36^ are shown in Table 9.

**Table 9:** Key size comparison results table.

Algorithm	Ours	Ref.^5^	Ref.^11^	Ref.^18^	Ref.^21^	Ref.^35^	Ref.^36^
Key size	10^240^	10^150^	1.54 × 10^96^	10^35^	2^448^	≈ 2^298^	2^128^

Table 10 shows the test values of the variance of the histogram and their comparison test results.

**Table 10:** Comparison test results table for the variance of histograms.

	Scale	Variance
Plain image		
Baboon	Gray	58,667.00
Ref.^5^ (Baboon)	Gray	58,542.00
Ref.^11^ (Baboon)	Gray	47,065.25
Encrypted image		
Baboon	Gray	217.83
Ref.^5^ (Baboon)	Gray	241.90
Ref.^11^ (Baboon)	Gray	233.35

From Tables 9 and 10, it can be seen that the algorithm has good encryption performance.

## Conclusion

In this study, we constructed a new 5D chaotic system using feedback control. The chaotic system can generate multiple bands and multiple wings in multiple directions, and the maximum Lyapunov exponent is approximately 15. Simultaneously, we applied the 5D multi-band multi-wing chaotic system to the hybrid image encryption algorithm for physical chaos encryption and algebraic encryption, and conducted a numerical simulation experiment on the hybrid encryption system. The experimental results verified the correctness of the encryption method. Therefore, the proposed encryption algorithm has promising potential applications in medical image encryption.

## Data Availability

The datasets used and/or analysed during the current study available from the corresponding author on reasonable request.
